# Cost-Effectiveness of Genomic Test-Directed Olaparib for Metastatic Castration-Resistant Prostate Cancer

**DOI:** 10.3389/fphar.2020.610601

**Published:** 2021-01-26

**Authors:** Dan Su, Bin Wu, Lizheng Shi

**Affiliations:** ^1^ Department of Pharmacy, the First Affiliated Hospital of University of Science and Technology of China, Hefei 230001, Anhui, China; ^2^Department of Pharmacy Medical Decision and Economic Group, Ren Ji Hospital, South Campus, School of Medicine, Shanghai Jiaotong University, Shanghai, China; ^3^ Department of Global Health Management and Policy, School of Public Health and Tropical Medicine, Tulane University, New Orleans, LA, United States

**Keywords:** olaparib, metastatic castration-resistant prostate cancer (mCRPC), next-generation sequencing, cost-effectiveness, partitioned survival model

## Abstract

**Purpose:** The effectiveness of poly (adenosine diphosphate–ribose) polymerase (PARP) inhibitor olaparib for metastatic castration-resistant prostate cancer (MCRPC) with multiple loss-of-function alterations in genes that are involved in DNA repair has been demonstrated. We aimed to evaluate the cost-effectiveness of genomic test-directed olaparib on MCRPC from the US payer perspective.

**Methods:** A partitioned survival model was adopted to project the disease course of MCRPC had at least one gene alteration in BRCA1, BRCA2 and ATM (Scenario A) and has alterations in any of all 15 prespecified genes (Scenario B) after next-generation sequencing test. The efficacy and toxicity data were gathered from the PROfound trial. Clinical probabilities related to survival were estimated from the reported survival probabilities in each PROfound group. Cost and health preference data were derived from the literature. The incremental cost-effectiveness ratio (ICER) was measured. Subgroup analysis and sensitivity analysis were performed for exploring the model uncertainties.

**Results:** Olaparib yielded an additional 0.063 and 0.068 of quality-adjusted life year (QALY) with the augmented cost of $7,382 and saved the cost of $ 1,980 compared to standard care in scenario A and B, respectively, which yielded an ICER of $116,903/QALY and a cost-saving option. The lower weekly cost related to olaparib treatment led to the dominant findings in scenario B. The varied results between scenario A and B could be partly explained by different the number need to screen for identifying eligible patients who could be administered with olaparib, which sharply augmented the costs of the olaparib arm in scenario A. Subgroup analysis and sensitivity analysis revealed the results were generally robust in both of two scenarios.

**Conclusion:** The genomic test-directed olaparib is a preferred option compared with standard care strategy for men with MCRPC who had any of all 15 prespecified genes.

## Introduction

Prostate cancer is one of the most common malignancies in men and a major cause of cancer deaths, accounting for 5.4% of the disease burden of all neoplasms, as reported by the GBD 2017 DALYs and HALE Collaborators, 2017 (2018). As the second leading cause of cancer death after lung cancer in the United States, the new cases and deaths of prostate cancer were estimated 191,930 and 33,330 for male Americans in 2020, respectively (Siegel et al., 2020). The recent overall incidence of prostate cancer has been decreasing over time due to the screening implementation, while the incidence of metastatic disease has been trending in the opposite direction (Attard et al., 2016). Metastatic castration-resistant prostate cancer (MCRPC) is a heterogeneous disease, whose annual incidence was about 36,100 in the United States (Scher et al., 2015). Although men with MCRPC could currently benefit from a wealth of effective treatment options, such as enzalutamide and abiraterone, the prognosis is still poor, whose median overall survival is varied from 9 to 13 months (He et al., 2020).

Deleterious aberrations in genes involved in repairing DNA damage, such as BRCA1, BRCA2 and ATM, were found in up to 30% of patients with prostate cancer (Bishop et al., 2019). Tumors with such gene alterations confer sensitivity to poly (adenosine diphosphate–ribose) polymerase (PARP) inhibition through multiple mechanisms, including trapping of PARP on DNA at sites of single-strand breaks (O’Sullivan et al., 2014). As an oral inhibitor of PARP, olaparib binds the catalytic domain of PARP1 leading to a reduction of PARylation and, therefore, to a defect in DNA repair (Lodovichi et al., 2019). Olaparib has been already used with success in pancreatic cancer and ovary cancer (Bao et al., 2016; Golan et al., 2019). The PROfound trial reported the efficacy and safety of olaparib for men with MCRPC who had any of all 15 prespecified genes that had direct or indirect role in homologous recombination repair and who had disease progression while receiving a new hormonal agent (de Bono et al., 2020; Hussain et al., 2020). The results revealed that olaparib markedly prolonged median progression-free survival (PFS) compared to the physician’s choice of enzalutamide or abiraterone in both the small sub-cohort anchoring with at least one of BRCA1, BRCA2 and ATM gene alterations (7.4 months vs. 3.6 months; hazard ratio [HR], 0.34; 95% confidence interval [CI], 0.25 to 0.47; *p* < 0.001) and big sub-cohort anchoring with at least one of 15 gene alterations (5.8 months vs. 3.5 months; HR, 0.49; 95% CI, 0.38 to 0.63; *p* < 0.001), and greater notably OS was also observed in both sub-cohorts (Hussain et al., 2020). Thus, the olaparib treatment seemed to be an attractive option for men who had disease progression while receiving enzalutamide or abiraterone and who had alterations in genes with a role in homologous recombination repair.

Due to the different prognosis of olaparib treatment in the two sub-cohorts and the high cost related to next-generation sequencing (NGS) test that should be used to prospectively identify patients with qualifying deleterious or suspected deleterious alterations, the following unclear question also needs to be elucidated: will the cost related to olaparib treatment compensate the cost of NGS test in both of two sub-cohorts? Will both of small and big sub-cohorts gain positive economic outcomes from the olaparib treatment. Herein, this analysis aimed to investigate the cost-effectiveness of olaparib in this context from the US payer perspective.

## Materials and Methods

### Analytical Overview

**FIGURE 1:**
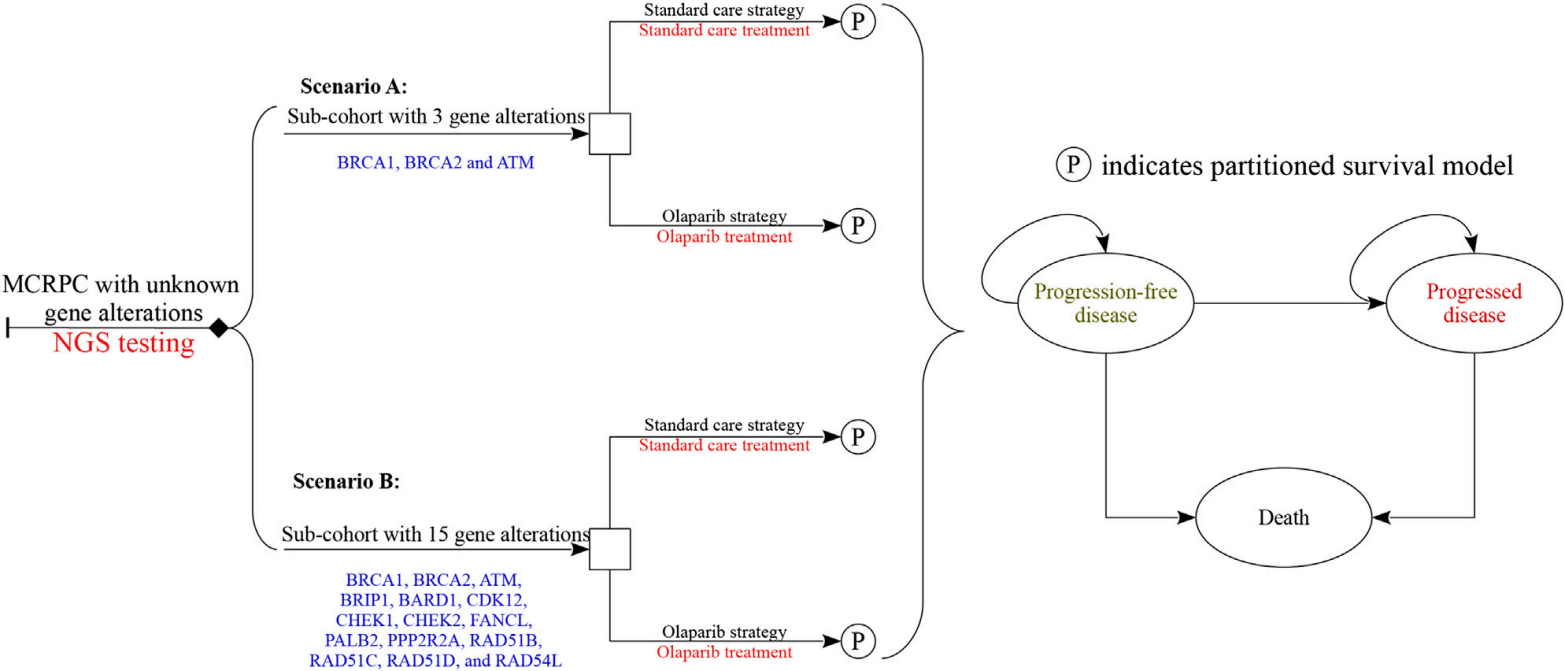
Diagrams of the decision trees combining partitioned survival model. Scenario **(A)** considered the targeted population who had at least one of the three gene alterations, and scenario **(B)** had any of all 15 gene alterations. In all strategies related to olaparib treatment, the cost of NGS test would be considered, and no cost related to genomic test was augmented in standard care strategy.

A mathematical model combining a decision tree and partitioned survival model was established to measure the clinical and economic outcomes of olaparib and standard care for men with MCRPC who had disease progression while receiving a new hormonal agent. The characteristics of hypothetical patients were based on the PROfound trial (de Bono et al., 2020). The decision trees included two scenarios after the results of NGS testing was obtained (Figure 1): patients with at least one of the BRCA1, BRCA2 and ATM gene alterations (scenario A) and the patients with at least one of the BRCA1, BRCA2, ATM, BRIP1, BARD1, CDK12, CHEK1, CHEK2, FANCL, PALB2, PPP2R2A, RAD51B, RAD51C, RAD51D, and RAD54L gene alterations (scenario B). Because the gene alterations need to be confirmed before olaparib was prescribed, the cost related to NGS testing would be incurred in the olaparib strategy. The partitioned survival model included the following health states: progression-free disease (PFD), progressed disease (PD), and death. In the three health states, OS was partitioned into alive with progression-free survival (PFS) and alive and with PD. The proportion of patients alive at cycle t (one-week cycle) was estimated by the area under the OS curve, and the proportion alive with PFS was estimated by the area under the PFS curve. The proportion alive and with PD was estimated by the difference between the OS and PFS curves. The proportions of patients with PFS and OS were based on the results of the PROfound trial (de Bono et al., 2020), which was validated by comparing predicted PFS and OS results with the observed data.

Cost and quality-adjusted life-year (QALY) were calculated with an annual discount rate of 3% in both the olaparib and standard care arms (Sanders et al., 2016). The incremental cost-effectiveness ratio (ICER) was expressed as the incremental cost per additional QALY gained between the two competing strategies. When the ICER was lower than the willingness-to-pay (WTP) threshold of $150,000/QALY, cost-effectiveness was assumed according to the recommendation (Neumann et al., 2014). We also estimated the incremental net health benefit (INHB) and incremental monetary benefit (INMB) based on the following formulas: INHB(*λ*) = (μ_E1_—μ_E0_)—(μ_C1_—μ_C0_)/λ = ΔE—ΔC/λ and INMB(*λ*) = (μ_E1_—μ_E0_)×λ—(μ_C1_—μ_C0_) = ΔE×λ—ΔC, where μ_Ci_ and μ_Ei_ were the cost and effectiveness of olaparib (i = 1) or standard care strategy (i = 0), respectively, and *λ* was the WTP threshold (Craig and Black, 2001).

### Clinical Data Inputs

PFS and OS in the olaparib and standard care groups were informed by the results of the PROfound trial (de Bono et al., 2020; Hussain et al., 2020) (at least the trial follow-up) and extrapolated over the model time horizon using standard statistical analyses described by Guyot et al. (Guyot et al., 2012). To avoid the effect of crossover from control therapy to olaparib on overall survival, the crossover-adjusted overall survival curves were adopted in the current analysis. R package “digitize” (version 0.0.4; https://github.com/tpoisot/digitize/) was used to gather the data points from the PFS and OS curves, and these data points were then used to fit the following parametric survival functions: Weibull, log-normal, log-logistic, exponential, generalized gamma, Gompertz and Royston/Parmar spline model and parametric mixture and nonmixture cure models. The eligible survival function was chosen based on the lowest value of the Akaike information criterion. The final survival functions of the olaparib and standard care in scenario A and B are shown in Table 1, and the goodness-of fit results are shown in Supplementary Appendix Tables S1 and S2. The validation plot, survival distribution, and HRs of the subgroups are shown in Supplementary Appendix Figures S1 and S2. Virtual patient-level data comprised event and censor times and were equal in number to the initial number at risk, which closely reproduced the digitized Kaplan–Meier curves. To reduce the uncertainty of exploring the long-term survival caused by the relatively low maturity of survival data in olaparib arm, the PFS and OS in olaparib arm after their observed time (18 and 24 months) were adjusted by multiplying the HRs for PFS and OS between the olaparib and standard care and the adjusted PFS and OS rate of standard care, respectively. The influences of the HRs for PFS and OS were checked with sensitivity and subgroup analyses. After disease progression, the data of patients who received subsequent treatment were collected from the PROfound trial (de Bono et al., 2020). Other key clinical inputs were summarized in Table 1.

**TABLE 1:** key model parameters: Baseline values, ranges, and distributions for sensitivity analysis.

Paramters	Expected value	Range	Distribution	References
Clinical inputs				(de Bono et al., 2020)
Survival model of standard care in scenario A				
Royston/Parmar spline model for PFS	gamma0: 3.965, gamma1: 5.800, gamma2: 2.247, gamma3: 1.103			
Gamma model for OS	Shape: 2.1689, rate: 0.1485			
Survival model of olaparib in scenario A				
Mixture cure model with log-logistic distribution for PFS	Theta: 0.097, shape: 2.383, scale: 2.889			
Gompertz model for OS	Shape: 0.06804, rate: 0.01423			
Survival model of standard care in scenario B				
Non-mixture cure model with lognormal distribution	Theta: 0.223, mean-log: 1.206, sd-log: 0.633			
Gamma model for OS	Shape: 2.0877, rate: 0.1387			
Survival model of olaparib in scenario B				
Gamma model for PFS	Shape: 1.540, rate: 0.161			
Royston/Parmar spline model for OS	gamma0: 4.838, gamma1: 0.887, gamma2: 0.873, gamma3: 1.294			
HR of PFS of between olaparib and standard care in scenario A	0.34	0.25–0.47	Lognormal: Log-mean = -1.079, Log-sd = 2.88	
HR of OS of between olaparib and standard care in scenario A	0.64	0.43–0.97	Lognormal: Log-mean = -0.446, Log-sd = 1.982	
HR of PFS of between olaparib and standard care in scenario B	0.49	0.38–0.63	Lognormal: Log-mean = -0.713, Log-sd = 2.752	
HR of OS of between olaparib and standard care in scenario B	0.67	0.49–0.93	Lognormal: Log-mean = -0.4, Log-sd = 2.187	
Proportion of receiving subsequent treatment				
Standard care	0.63	0.476–0.793	Beta: *α* = 5.9, *β* = 3.4	
Olaparib	0.35	0.264–0.44	Beta: *α* = 10.4, *β* = 19.1	
Utility inputs				
PFD	0.76	0.65–0.87	Beta: *α* = 44, *β* = 13.9	(Bremner et al., 2007; Barqawi et al., 2019)
PD	0.37	0.33–0.41	Beta: *α* = 207.1, *β* = 352.6	(Bremner et al., 2007; Barqawi et al., 2019)
Disutility due to grade 1–2 AEs	0.01	0.008–0.02	Beta: *α* = 18, *β* = 1,283.2	(Amdahl et al., 2016)
Disutility due to grade ≥3 AEs	0.16	0.11–0.204	Beta: *α* = 36, *β* = 193	(Amdahl et al., 2016)
Cost inputs				
Olaparib 600 mg per day	246.80	123.4–246.8	Fixed	(Red book online, 2020)
Abiraterone + prednisone per week	2,395	2,156–2,634	Gamma: *α* = 46,961, *β* = 0.051	(Barqawi et al., 2019)
Enzalutamide per week	2,559	2,304–2,814	Gamma: *α* = 50,176, *β* = 0.051	(Barqawi et al., 2019)
Salvage therapy with docetaxel per month	1,462	1,096–1,827	Gamma: *α* = 5,848, *β* = 0.25	(Barqawi et al., 2019)
Salvage therapy with enzalutamide per month	9,821	7,366–12,277	Gamma: *α* = 39,284, *β* = 0.25	(Massoudi et al., 2017)
Salvage therapy with abiraterone per month	9,597	7,198–11,996	Gamma: *α* = 38,388, *β* = 0.25	(Massoudi et al., 2017)
Salvage therapy with cabazitaxel per month	14,864	11,148–18,580	Gamma: *α* = 59,456, *β* = 0.25	(Massoudi et al., 2017)
Supportive care per day	190	142–237	Gamma: *α* = 4, *β* = 49.921	(Massoudi et al., 2017)
Terminal care	36,403	27,117–45,689	Gamma: *α* = 142,757, *β* = 0.255	(Sathianathen et al., 2019)
Follow-up per week	146	109–182	Gamma: *α* = 591, *β* = 0.247	(Wu et al., 2020)
Managing vomiting per event (Grade ≥3)	2,638	1,978–3,297	Gamma: *α* = 10,552, *β* = 0.25	(Soto-Perez-de-Celis et al., 2019)
Managing backpain per event (Grade ≥3)	11,815	10,633–12,996	Gamma: *α* = 231,667, *β* = 0.051	(Barqawi et al., 2019; Soto-Perez-de-Celis et al., 2019)
Managing anemia per event (Grade ≥3)	145	1–2,099	Gamma: *α* = 39, *β* = 3.69	(Barqawi et al., 2019; Soto-Perez-de-Celis et al., 2019)
Managing fatigue per event (Grade ≥3)	858	729–986	Gamma: *α* = 5,720, *β* = 0.15	(Barqawi et al., 2019; Soto-Perez-de-Celis et al., 2019)

HR, hazard ratio; PFS, progression-free survival; PFD, progression-free disease; PD, progressed disease; OS, overall survival.

### Cost and Utility Inputs

Direct medical costs were considered from a United States payer perspective, which was reported in 2019 United States dollars. The direct medical costs considered were as follows: drug acquisition costs, costs attributed to the patient’s health state, costs for the management of AEs, and costs of end-of-life care (Table 1). When necessary, cost estimates were adjusted for inflation to 2019 values using the Medical-care inflation calculator. (2020).

Based on the PROfound trial (de Bono et al., 2020), olaparib group received the standard dose of olaparib tablets (300 mg twice daily). Patients assigned to the standard care group received enzalutamide (160 mg once daily) or abiraterone (1,000 mg once daily, plus prednisone at a dose of 5 mg twice daily). The proportion of receiving enzalutamide was 54% (range: 45%–64%). Treatment continued until disease progression or unacceptable toxicity. The cost related to olaparib, enzalutamide and abiraterone treatments were collected from the literature and public database (CMS Drug Cost, 2019; Barqawi et al., 2019). In the United States, the price of olaparib, enzalutamide and abiraterone were discounted at 15% to account for contract pricing (Barqawi et al., 2019). After disease progressed, 35.2% in olaparib arm and 63.4% in the standard care arm received subsequent active therapy. The proportion of subsequent treatment regimens were extracted from the PROfound trial (Supplementary Appendix Table S3) (de Bono et al., 2020). The length of second-line treatment was derived from a previous study (Pollard et al., 2017). Other people who did not receive subsequent active therapy were assumed to be administered with supportive care. The costs of subsequent treatment regimens and supportive care were gathered from a cost study among United States patients with MCRPC(Massoudi et al., 2017). The cost related to follow-up was $146 per week (Wu et al., 2020). Each prostate cancer death event would augment the cost of $36,403 (Barqawi et al., 2019). The analysis included the costs related to managing grade ≥3 adverse events (AEs), which were extracted from the literature (Barqawi et al., 2019; Soto-Perez-de-Celis et al., 2019). Because identifying the eligible patients with gene alterations is necessary before the administration olaparib, the cost related to FoundationOne CDx next-generation sequencing test would be considered in the olaparib strategy (Gong et al., 2018). After the gene alterations screening, the incurred costs in those with negative gene alterations would be added into the olaparib strategy. The proportions of one or more of the 15 prespecified genes was 28%, where the proportions of BRCA1/2 and ATM gene alterations was 63% (de Bono et al., 2020). In the standard care strategy, no cost related to sequencing test was included.

Each Markov health state was assigned a health utility preference on a scale of 0 (death) to 1 (perfect health). We assumed the health utility preference was only associated with the disease status. The PFD and PD states related to MCRPC were 0.76 and 0.37 (Bremner et al., 2007; Barqawi et al., 2019), respectively. The disutility values due to AEs were included in this analysis (Amdahl et al., 2016). All AEs were assumed to be incurred in the first cycle. The duration-adjusted disutility was subtracted from the baseline PFD utility.

### Analysis

To evaluate the robustness of the base-case result, one-way and probabilistic sensitivity analyses (PSA) were conducted. One-way sensitivity analyses were conducted for all parameters, and the estimated range of each parameter was based on either the reported or estimated 95% confidence intervals in the referenced studies or determined by assuming a 25% change from the base-case value (Table 1). In the PSA, a Monte Carlo simulation with 10,000 iterations was generated by simultaneously sampling the key model parameters from the prespecified distributions. A gamma distribution was selected for the cost parameters, a log-normal distribution for the HRs, and a beta distribution for probability, proportion and preference value parameters. Based on the data from 10,000 iterations, a cost-effectiveness acceptability curve (CEAC) was created to represent the likelihood that atezolizumab plus bevacizumab would be considered cost-effective at various WTP levels for health gains (QALYs). Subgroup analyses were performed for the prespecified subgroups that were reported in the trials by varying the HRs for PFS except the region subgroups. This analysis was based on the EQUATOR Reporting Guidelines (CHEERS, Supplementary Appendix Table S4).

## Results

### Base-Case Analysis

In comparison with standard care, olaparib produced a marginal 0.100 overall life years and 0.063 QALYs in scenario A (Table 2), and 0.125 overall life years and 0.068 QALYs in scenario B. The olaparib treatment augmented the total cost of $7,382 compared with standard care in scenario A, which included the additional costs of $1,980 in the PFD state. In scenario B, the olaparib treatment saved the total cost of $6,950 in comparison with standard care, which was mainly driven by the reduced costs of $12,166 in the PFD state. The olaparib treatment led to an ICER of $116,903/QALY in scenario A and presented as a dominant option in scenario B, respectively. The INHB and INMB were 0.01 QALYs and $2,090 in scenario A and 0.114 QALYs and $17,109 in scenario B at the threshold of $150,000/QALY, respectively.

**TABLE 2:** Summary of cost ($) and outcome results in base-case analysis.

Strategy	Cost outcomes		Health outcomes		Incremental cost per QALY^a^	INHB^a^	INMB^a^
	Total cost	Cost in the PFD state	Overall LYs	QALYs			
Scenario A: 3 gene alterations							
Standard care	17,243	10,055	0.221	0.109	–	–	–
Olaparib	24,626	12,035	0.321	0.173	116,903	0.01	2,090
Scenario **B**: 15 gene alterations							
Standard care	55,476	44,261	0.540	0.313	–	–	–
Olaparib	48,526	32,096	0.665	0.380	Dominance	0.114	17,109

^a^Comparing with standard care and Included the NGS cost ($5,800).

### Sensitivity Analyses

**FIGURE 2:**
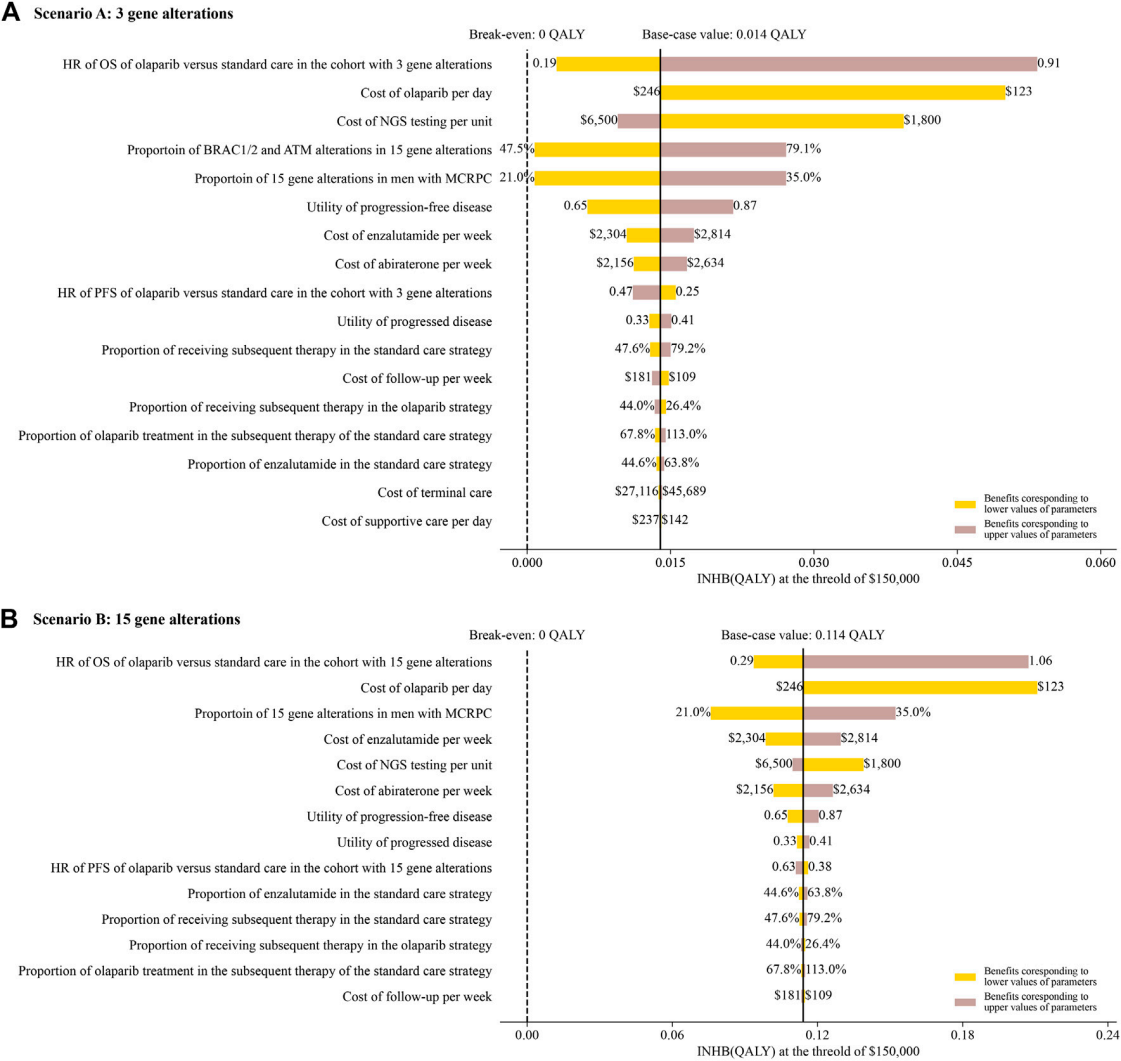
Tornado diagram of one-way sensitivity analyses of olaparib vs. standard care in order of magnitude of the association in scenario **A** and **B**.

The one-way sensitivity analyses revealed that the model outcomes were most sensitive to the HR of OS and the cost of olaparib in both of scenario A and B (Figures 2A,B). Other parameters, such as the cost of standard care and utility scores, had medium and small impact on the outcome. However, none of the adjustments of these parameters could drive the INHBs to be lower than the break-even value (0 QALY).

**FIGURE 3:**
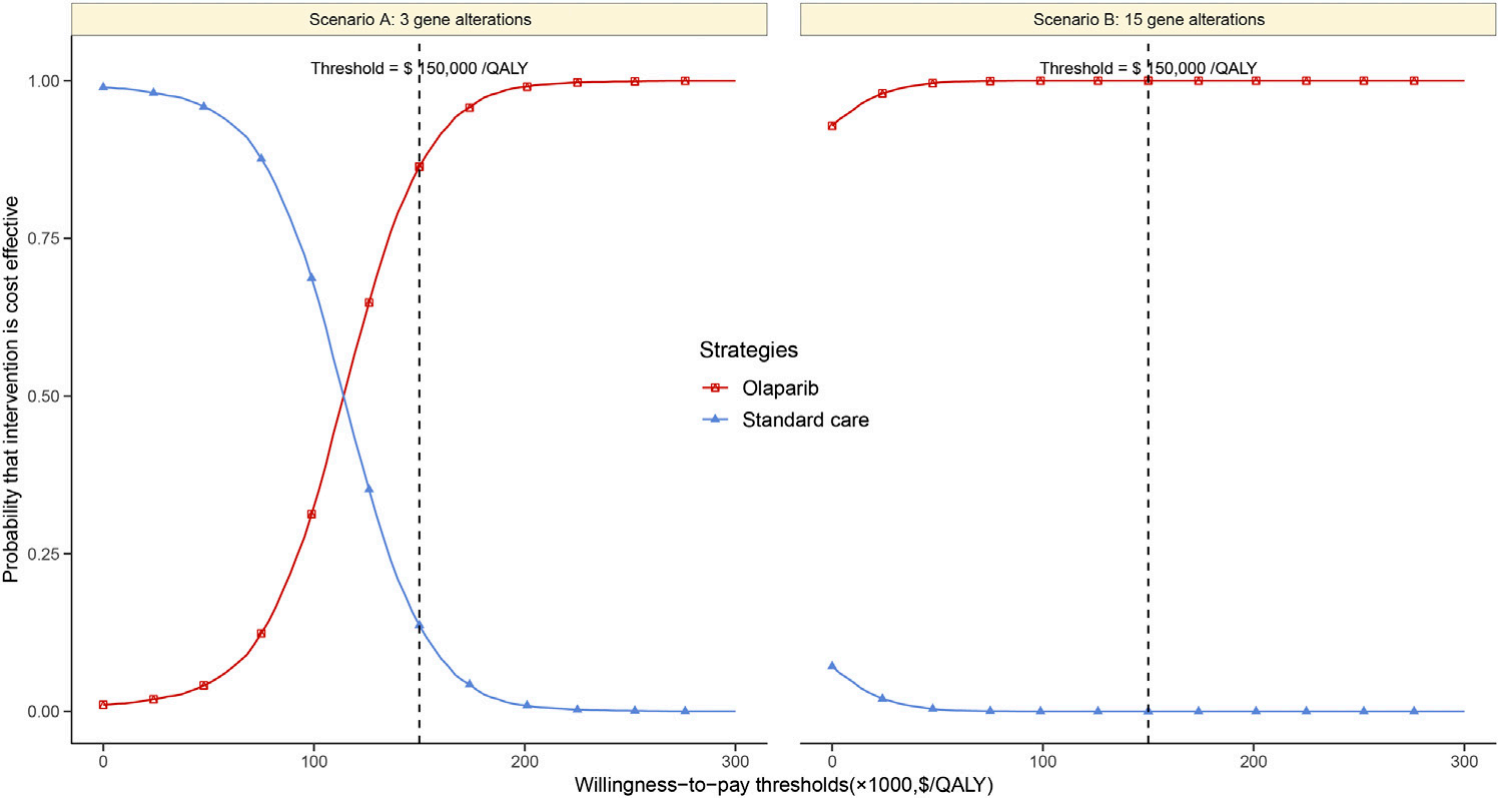
Cost-effectiveness acceptability curves of olaparib vs. standard care groups in scenario **(A)** and **(B)**.

Based on the Monte Carlo simulation of 10,000 patients, the CEAC showed nearly 86% and 100% probabilities of olaparib being a cost-effective strategy in scenario A and B at the threshold of $150,000/QALY gained (Figure 3).

### Subgroup Analyses

**FIGURE 4:**
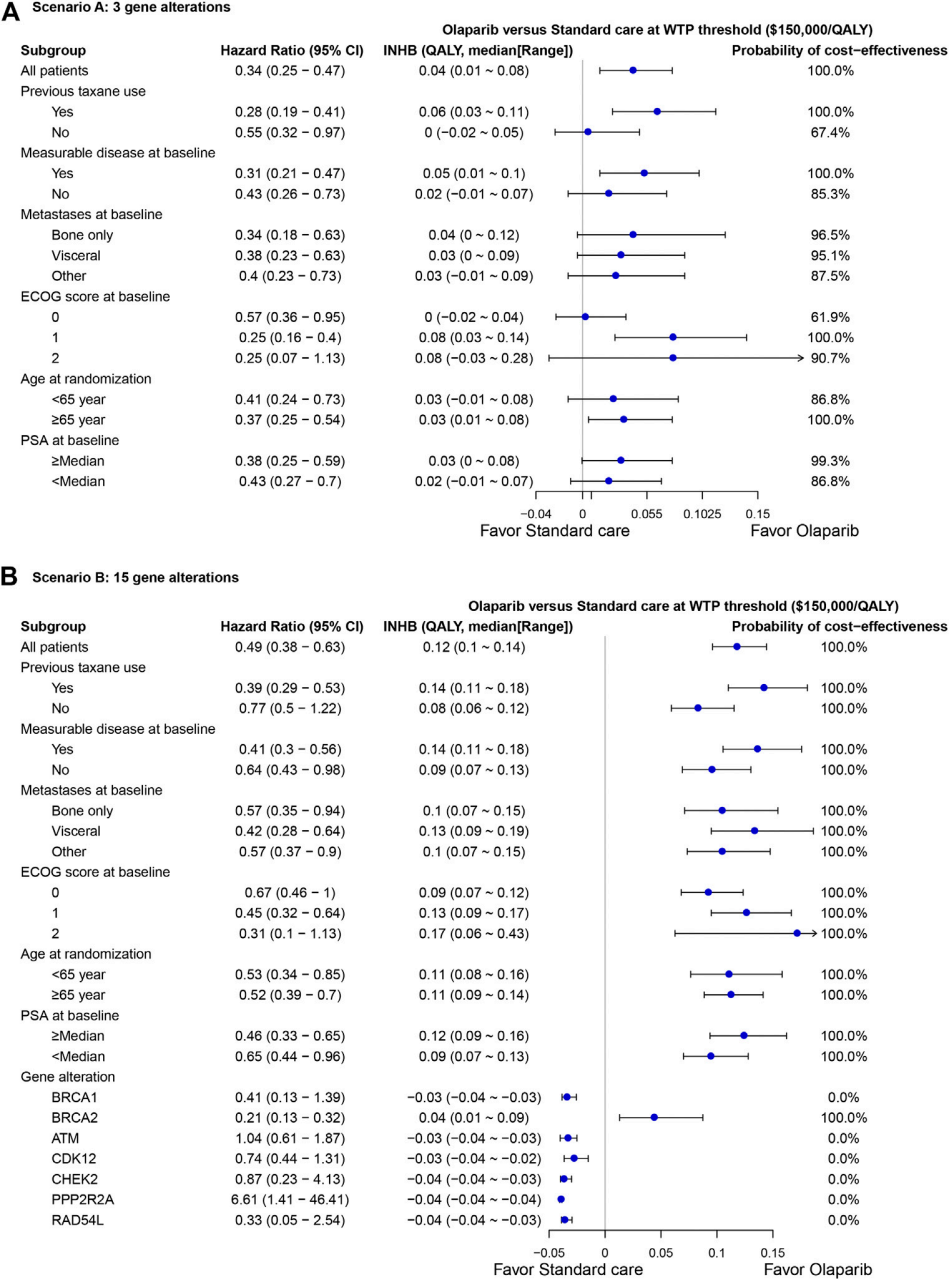
Subgroup analysis of incremental net health benefit (INHB) and probabilities of cost-effectiveness by varying the hazard ratios (HRs) of PFS in scenario **(A)** and **(B)**. The vertical line indicates the point of no effect (INHB = 0), the blue circle indicates the median INHB, and the black line indicates the ranges of INHB adjusted by the HRs.

The subgroup analysis by varying the HRs of PFS suggested that olaparib treatment gained the positive trend of gaining INHB in all the subgroups in scenario A at the threshold of $150,000/QALY (Figure 4A), where the probabilities of cost-effectiveness were lower than61% in all subgroups. In scenario B, the olaparib treatment strategy gained a positive trend of gaining INHB in all the subgroups except the BRCA1, ATM, CDK12, CHEK2, PPP2R2A and RAD54 L gene alterations (Figure 4B). The economic outcomes in patients anchoring the BRCA2 mutations were more favorable than the res of alterations, such as BRCA1 and ATM alterations.

## Discussion

Based on the results of PROfound trial (de Bono et al., 2020), the current evaluation demonstrated olaparib therapy for the sub-cohort with MCRPC anchoring at least one of 15 gene alterations could be a cost-saving option even when the cost of NGS testing for gene alterations was considered. This preferred finding also kept in the subgroup and sensitivity analyses. Except for the subgroup with BRCA1, ATM, CDK12, CHEK2, PPP2R2A and RAD54 L alterations, the rest of subgroups were absolutely favored to the olaparib treatment because it yielded achieved 100% probabilities of cost-effectiveness and its ranges of INHBs were all greater than zero. The weekly cost of olaparib was relatively 39% lower than standard care, which could mainly explain the preferred finding. However, when olaparib was only prescribed for MCRPC with at least one of BRCA1/2 and ATM gene alterations, it will become to be cost-effective because the augmented cost would become positive with additional QALAYs, which yielded an ICER to be lower than $150,000/QALY. In this small sub-cohort, all of subgroups could gain positive INHBs compared to standard care. The following potential two reasons might explain these varied result between scenario A and B: 1) In the small cohort, the cost in PFD state of the olaparib arm was about 20% higher than the standard care arm, which might augment the cost related to olaparib treatment due to its longer treatment duration. Because the PROfound trial showed that the patients anchoring alterations except BRCA1/2 and ATM alterations have the comparable overall survival between the olaparib and standard care strategies (de Bono et al., 2020), it could be indicated that these patients could gain comparable health outcomes by consuming less health resources if the relatively lower-cost olaparib was administered; 2) Because the proportion of MCRPC with at least one of BRCA1/2 and ATM gene alterations was about 18% in the whole MCRPC(de Bono et al., 2020), the number need to screen was 5.6 for identifying eligible patients who could be administered with olaparib, which was two greater than MCRPC anchoring any of all 15 gene alterations. Therefore, the cost related to NGS testing sharply augmented the costs in PFD state of the olaparib arm, which could not be compensated by the lower weekly costs of olaparib in this small sub-cohort as the sub-cohort with MCRPC anchoring any of all 15 gene alterations done. This reason could be proved by the subgroup analysis in scenario B, which found economic outcomes were negative for the lower prevalence of alterations (BRCA1, ATM, CDK12, CHEK2, PPP2R2A and RAD54L) because of the higher number need to screen per individual gene alteration, and positive for the higher prevalence of alterations (BRCA2). Because the cost related to the gene alteration screening is a substantial factor, lowing the cost of NGS testing might drive the INHBs of olaparib vs. standard care to be more favorable as shown by one-way sensitivity analysis.

Because of the fact that prostate cancer is the most common cancer in Western countries and the various novel therapy alternatives, the cost of this illness will be substantial and rising. In this context, the documented possible savings or limitation of augmenting costs should be encouraged. Both abiraterone and enzalutamide are recommended as the standard care for the treatment of MCRPC for patients with no prior docetaxel treatment (Mohler et al., 2019). However, the economic evaluations showed both of them were unfavorable options compared to other therapies because their ICERs were above or around a regularly used WTP threshold for oncology drugs with a low probability of cost-effectiveness (Grochtdreis et al., 2018). To our best of knowledge, this is the first analysis to measure the cost-effectiveness of olaparib treatment and standard care for men with MCRPC anchoring any of all 15 prespecified gene alterations by synthesizing the latest clinical evidence through a modeling approach. The findings of one-way sensitivity analysis also demonstrated that the cost related to olaparib could play an active role in driving the economic outcomes, which suggested that lowing the cost related to olaparib could drive the INHBs of olaparib vs. standard care to be more optional in both the two sub-cohorts.

The subgroup analysis found that olaparib would achieve more INHBs compared to standard care in the subgroups with more favorable HR of PFS. It could be mainly explained by the fact that longer duration in the PFD state was associated with the higher health outcomes and lower costs in PFD state of the olaparib arm. However, longer duration in the progressed disease state was associated with the greater total costs of the olaparib arm than the standard care arm due to the crossover effect that patients in olaparib arm would receive relatively expensive abiraterone and enzalutamide treatment after disease progressed, and those in standard care arm would receive relatively cheaper olaparib treatment. When the HR of PFS was fixed, this crossover effect partly explained the results of one-way sensitivity analysis that more favorable HR of OS would result in less INHBs because of the relatively higher cost in the progressed disease state, which could not be compensated by the longer duration in the progressed disease state due to its relatively lower health utility preference. For saving more money and gain more health benefits, the above findings indicated that olaparib should be placed in front of hormonal agents for MCRPC with any of all 15 prespecified genes after disease progression while receiving a new hormonal agent.

There are several weaknesses in the study. Firstly, due to the lack of data, other PARP inhibitors, such as rucaparib and niraparib, were not included in this analysis, which have shown positive results in a small number of patients (Ratta et al., 2020). The present study needs to be updated when new clinical data become available. Secondly, by using the well-accepted approach, we explored the survival probabilities beyond the observation time of the PROfound study through the fitting of survival functions to the observed survival data. Thirdly, we did not project the budget impact of olaparib on society. A wide prescription of olaparib might mitigate the financial burden because its relatively lower weekly costs compared to standard care. Especially when olaparib is prescribed for MCRPC anchoring at least one of 15 gene alterations, the saved cost related to olaparib could compensate for the cost of NGS testing. Fourthly, because this analysis was conducted from the US context which had varied cost estimates and treatment patterns in comparison with other countries, the influence of cost and effects estimates should be examined when transferring economic data between countries (Knies et al., 2009). Finally, the costs of grade 1/2 adverse events were excluded from the present study. However, because the probability of grade 1/2 adverse events in the olaparib arm is comparable with the standard care arm (44% vs. 50%), this weakness may not be a major one. This speculation also could be implied by the findings in one-way sensitivity analysis, which suggested the costs related to adverse events only have a paucity of impact.

These estimates suggested that olaparib treatment was cost-effective option for men with MCRPC who had any of all 15 prespecified genes that had a direct or indirect role in homologous recombination repair and who had disease progression while receiving a new hormonal agent. These findings might be helpful in making a rational decision for the treatment of MCRPC. Because of the methodological flaws in the current analysis, more quality clinical and economic real-world data are needed in the future; we believe that this focus will provide more sound evidence as a framework for determining the value of different therapeutic alternatives in oncology.

## Disclaimer

The views expressed are those of the authors.

## Data Availability Statement

The original contributions presented in the study are included in the article/Supplementary Material, further inquiries can be directed to the corresponding authors.

## Ethics Statement

This study was based on a literature review and modeling techniques; this study did not require approval by an institutional research ethics board.

## Author Contributions

LS and BW designed the study. DS and BW collected the data and performed the economic analysis. BW wrote the first draft of the manuscript, which was critically revised by LS.

## Funding

This work was sponsored by unrestricted grants from the National Natural Science Foundation of China (NO. 7172810).

## Conflict of Interest

The authors declare that the research was conducted in the absence of any commercial or financial relationships that could be construed as a potential conflict of interest.

## Abbreviations

HR, hazard ratio; PFS, progression-free survival; PFD, progression-free disease; PD, progressed disease; OS, overall survival.
