# Supplementary material for: Cost-Effectiveness of Genomic Test-Directed Olaparib for Metastatic Castration-Resistant Prostate Cancer
Source: Front Pharmacol. 2021 Jan 26;11:610601. doi: 10.3389/fphar.2020.610601 (PMC7870786; doi:10.3389/fphar.2020.610601)
Supplement: Supplementary file 1 [file table1.pdf]

Appendix Table 1. Estimated parameters and AIC values from each survival model in MCRPC with at least 1 of the BRCA1/2 and ATM gene alterations.

Strategies	Distributions	Parameters	PFS					OS				
			est	L95%	U95%	se	AIC	est	L95%	U95%	se	AIC
Standard care	Weibull	shape	1.3230	1.1118	1.5743	0.1174	341.24	1.7122	1.3273	2.2086	0.2224	343.64
		scale	4.8403	4.0321	5.8105	0.4512		15.5116	12.9653	18.5579	1.4191	
	Gamma	shape	1.9376	1.4242	2.6361	0.3044	334.60	2.1689	1.4905	3.1563	0.4152	343.54
		rate	0.4426	0.3055	0.6414	0.0837		0.1485	0.0903	0.2444	0.0377	
	Exp	rate	0.2140	0.1687	0.2714	0.0260	347.68	0.0536	0.0400	0.0717	0.0080	355.43
	Log-logistic	shape	2.2873	1.8834	2.7777	0.2267	320.89	2.0805	1.6156	2.6792	0.2685	343.85
		scale	3.1831	2.6873	3.7704	0.2750		12.3787	10.1617	15.0793	1.2464	
	Log-normal	meanlog	1.2056	1.0390	1.3723	0.0850	319.57	2.5203	2.2953	2.7453	0.1148	345.63
		sdlog	0.7453	0.6273	0.8855	0.0655		0.8811	0.7054	1.1004	0.0999	
	Gompertz	shape	0.0130	-0.0587	0.0848	0.0366	349.55	0.1065	0.0430	0.1700	0.0324	347.13
		rate	0.2052	0.1466	0.2872	0.0352		0.0252	0.0139	0.0456	0.0076	
	Royston/Parmar spline model (0 knot)	gamma0	-2.0863	-2.5573	-1.6153	0.2403	341.24	-4.6964	-5.8358	-3.5569	0.5814	343.64
		gamma1	1.3230	1.0929	1.5530	0.1174		1.7131	1.2770	2.1492	0.2225	
	Royston/Parmar spline model (1 knot)	gamma0	-3.1814	-3.9646	-2.3982	0.3996	317.19	-4.8526	-6.6949	-3.0104	0.9399	345.59
		gamma1	3.1287	2.2556	4.0018	0.4455		1.8405	0.6033	3.0777	0.6312	
		gamma2	0.4306	0.2527	0.6086	0.0908		0.0440	-0.3485	0.4364	0.2002	
	Royston/Parmar spline model (2 knot)	gamma0	-3.9647	-5.1056	-2.8237	0.5821	312.19	-4.8610	-6.7914	-2.9306	0.9849	347.60
		gamma1	5.7999	3.3308	8.2690	1.2598		1.8616	0.3908	3.3325	0.7504	
		gamma2	2.2473	0.6235	3.8710	0.8285		0.1017	-1.8045	2.0079	0.9726	
		gamma3	-1.1034	-2.1708	-0.0360	0.5446		-0.1181	-3.5425	3.3062	1.7471	
	Mixture cure model (Weibull)	theta	0.0682	0.0237	0.1806	NA	331.37	0.1943	0.0287	0.6632	NA	345.08
		shape	1.6121	1.3462	1.9306	0.1483		1.9085	1.3484	2.7012	0.3383	
		scale	4.0948	3.4720	4.8293	0.3447		12.4412	8.1577	18.9739	2.6790	
	Mixture cure model (Gamma)	theta	0.0708	0.0251	0.1840	NA	324.61	0.0227	0.0000	1.0000	NA	345.54
		shape	2.6594	1.9132	3.6966	0.4468		2.1985	1.2986	3.7219	0.5905	
		rate	0.7348	0.4965	1.0876	0.1470		0.1543	0.0512	0.4653	0.0869	
	Mixture cure model (Exp)	theta	0.0282	0.0022	0.2774	NA	348.89	0.0001	0.0000	1.0000	NA	357.43
		rate	0.2336	0.1741	0.3136	0.0351		0.0536	0.0400	0.0718	0.0080	
		theta	0.0569	0.0140	0.2040	NA	319.84	0.0006	0.0000	1.0000	NA	345.85

	Mixture cure model (Log-logistic)	shape	2.5730	2.0649	3.2063	0.2888		2.0811	1.6151	2.6817	0.2692	
		scale	2.9533	2.4895	3.5036	0.2575		12.3721	10.1283	15.1131	1.2632	
	Mixture cure model (Log-normal)	theta	0.0630	0.0187	0.1920	NA	316.69	0.0004	0.0000	1.0000	NA	347.64
		meanlog	1.1024	0.9398	1.2650	0.0830		2.5199	2.2937	2.7460	0.1154	
		sdlog	0.6444	0.5317	0.7810	0.0632		0.8809	0.7051	1.1005	0.1000	
	Mixture cure model (Gompertz)	theta	0.0608	0.0192	0.1763	NA	344.68	0.3026	0.1785	0.4642	NA	344.48
		shape	0.1353	0.0444	0.2261	0.0464		0.2156	0.1219	0.3092	0.0478	
		rate	0.1747	0.1201	0.2543	0.0335		0.0217	0.0103	0.0455	0.0082	
	Non-mixture cure model (Weibull)	theta	0.0657	0.0237	0.1692	NA	326.03	0.0763	0.0000	0.9944	NA	345.35
		shape	1.9131	1.5945	2.2953	0.1778		1.8987	1.2611	2.8588	0.3964	
		scale	6.2870	4.8231	8.1953	0.8503		22.7266	3.2357	159.6229	22.6027	
	Non-mixture cure model (Gamma)	theta	0.0739	0.0276	0.1834	NA	321.15	0.0013	0.0000	1.0000	NA	345.45
		shape	2.8450	2.0678	3.9145	0.4632		1.9512	1.0851	3.5085	0.5841	
		rate	0.5192	0.2970	0.9078	0.1480		0.0420	0.0006	3.0983	0.0922	
	Non-mixture cure model (Exp)	theta	0.0000	0.0000	1.0000	NA	349.85	0.0000	0.0000	1.0000	NA	357.64
		rate	0.0072	0.0003	0.1591	0.0114		0.0011	0.0000	1.0685	0.0039	
	Non-mixture cure model (Log-logistic)	theta	0.0705	0.0234	0.1934	NA	321.63	0.0052	0.0000	1.0000	NA	345.40
		shape	2.3788	1.8732	3.0209	0.2900		1.8745	1.2301	2.8565	0.4029	
		scale	4.8617	3.4780	6.7959	0.8308		33.8165	2.3901	478.4489	45.7152	
	Non-mixture cure model (Log-normal)	theta	0.0641	0.0182	0.2021	NA	316.21	0.0000	0.0000	1.0000	NA	345.68
		meanlog	1.6210	1.2209	2.0210	0.2041		5.7198	1.3034	10.1361	2.2533	
		sdlog	0.7406	0.5704	0.9616	0.0987		1.5745	0.8958	2.7674	0.4531	
	Non-mixture cure model (Gompertz)	theta	0.0536	0.0171	0.1557	NA	342.84	0.3031	0.1733	0.4742	NA	344.73
		shape	0.2398	0.1404	0.3392	0.0507		0.2415	0.1406	0.3423	0.0515	
		rate	0.0508	0.0293	0.0879	0.0142		0.0120	0.0054	0.0266	0.0049	
Olaparib	Weibull	shape	1.2375	1.0659	1.4366	0.0942	510.81	1.6459	1.3558	1.9980	0.1628	572.03
		scale	5.1008	4.3439	5.9895	0.4180		16.1037	13.9764	18.5548	1.1641	
	Gamma	shape	1.6070	1.2513	2.0638	0.2051	505.42	2.0877	1.5632	2.7883	0.3082	571.45
		rate	0.3419	0.2481	0.4710	0.0559		0.1387	0.0948	0.2031	0.0270	
	Exp	rate	0.1977	0.1622	0.2410	0.0200	515.74	0.0537	0.0428	0.0673	0.0062	590.64
	Log-logistic	shape	2.0079	1.7054	2.3641	0.1673	487.41	2.0427	1.6839	2.4780	0.2013	571.78
		scale	3.3099	2.8374	3.8611	0.2601		12.5654	10.7302	14.7146	1.0122	
	Log-normal	meanlog	1.2434	1.0678	1.4190	0.0896	504.98	2.5313	2.3546	2.7080	0.0902	574.72
		sdlog	0.9644	0.8348	1.1140	0.0710		0.8896	0.7500	1.0551	0.0774	
	Gompertz	shape	-0.0072	-0.0716	0.0571	0.0328	517.69	0.0867	0.0431	0.1302	0.0222	578.31
		rate	0.2022	0.1527	0.2678	0.0290		0.0277	0.0178	0.0432	0.0063	

	Royston/Parmar spline model (0 knot)	gamma0	-2.0163	-2.3912	-1.6414	0.1913	510.81	-4.5740	-5.4348	-3.7131	0.4392	572.03
		gamma1	1.2375	1.0528	1.4221	0.0942		1.6459	1.3268	1.9650	0.1628	
	Royston/Parmar spline model (1 knot)	gamma0	0.4276	-0.8065	1.6616	0.6296	495.38	-4.9801	-6.4818	-3.4783	0.7662	573.55
		gamma1	4.5502	2.7863	6.3140	0.8999		1.9921	0.9215	3.0627	0.5462	
		gamma2	0.1391	0.0674	0.2107	0.0366		0.0683	-0.1293	0.2660	0.1009	
	Royston/Parmar spline model (2 knot)	gamma0	-4.0997	-5.6912	-2.5081	0.8120	484.38	-4.8608	-6.4165	-3.3051	0.7937	575.39
		gamma1	0.6861	-0.5892	1.9614	0.6507		1.8165	0.4917	3.1414	0.6760	
		gamma2	-0.5776	-0.8294	-0.3258	0.1285		-0.2303	-1.4961	1.0355	0.6458	
		gamma3	0.8065	0.4912	1.1218	0.1609		0.3744	-1.2454	1.9943	0.8265	
	Mixture cure model (Weibull)	theta	0.1156	0.0589	0.2142	NA	493.98	0.1491	0.0238	0.5575	NA	573.31
		shape	1.5710	1.3453	1.8346	0.1243		1.7913	1.3873	2.3128	0.2335	
		scale	3.9180	3.3718	4.5528	0.3001		13.4946	9.6990	18.7754	2.2739	
	Mixture cure model (Gamma)	theta	0.1148	0.0578	0.2151	NA	489.84	0.0349	0.0000	0.9995	NA	573.42
		shape	2.2789	1.7288	3.0040	0.3212		2.1419	1.4294	3.2095	0.4420	
		rate	0.6460	0.4539	0.9195	0.1163		0.1483	0.0656	0.3354	0.0617	
	Mixture cure model (Exp)	theta	0.0550	0.0119	0.2188	NA	515.62	0.0001	0.0000	1.0000	NA	592.64
		rate	0.2294	0.1758	0.2993	0.0311		0.0537	0.0428	0.0674	0.0062	
	Mixture cure model (Log-logistic)	theta	0.0966	0.0394	0.2181	NA	483.38	0.0001	0.0000	1.0000	NA	573.78
		shape	2.3828	1.9591	2.8982	0.2380		2.0431	1.6839	2.4788	0.2015	
		scale	2.8887	2.4616	3.3899	0.2358		12.5642	10.7230	14.7215	1.0158	
	Mixture cure model (Log-normal)	theta	0.0498	0.0073	0.2714	NA	505.88	0.0001	0.0000	1.0000	NA	576.73
		meanlog	1.1542	0.9340	1.3743	0.1123		2.5312	2.3543	2.7081	0.0902	
		sdlog	0.8984	0.7472	1.0803	0.0845		0.8894	0.7499	1.0549	0.0774	
	Mixture cure model (Gompertz)	theta	0.1028	0.0487	0.2039	NA	509.48	0.2291	0.1206	0.3919	NA	576.91
		shape	0.1420	0.0600	0.2240	0.0419		0.1459	0.0845	0.2072	0.0313	
		rate	0.1781	0.1298	0.2444	0.0288		0.0279	0.0170	0.0457	0.0070	
	Non-mixture cure model (Weibull)	theta	0.1091	0.0551	0.2046	NA	488.77	0.0716	0.0005	0.9214	NA	573.34
		shape	1.8211	1.5581	2.1284	0.1449		1.8398	1.3719	2.4672	0.2755	
		scale	5.7320	4.5424	7.2332	0.6803		24.0630	6.4741	89.4368	16.1183	
	Non-mixture cure model (Gamma)	theta	0.1078	0.0519	0.2103	NA	486.26	0.0083	0.0000	0.9998	NA	573.37
		shape	2.4552	1.8779	3.2099	0.3357		1.9560	1.2581	3.0408	0.4403	
		rate	0.4603	0.2792	0.7587	0.1174		0.0507	0.0042	0.6181	0.0647	
		theta	0.0000	0.0000	1.0000	NA	517.71	0.0000	0.0000	1.0000	NA	593.16

	Non-mixture cure model (Exp)	rate	0.0120	0.0004	0.3769	0.0211		0.0016	0.0002	0.0150	0.0018	
	Non-mixture cure model (Log-logistic)	theta	0.1052	0.0483	0.2140	NA		0.0108	0.0000	0.9957	NA	
		shape	2.2450	1.8233	2.7643	0.2383	484.18	1.8498	1.3419	2.5500	0.3030	573.36
		scale	4.5596	3.3843	6.1429	0.6934		31.8498	5.9669	170.0055	27.2158	
	Non-mixture cure model (Log-normal)	theta	0.0124	0.0003	0.3750	NA		0.0000	0.0000	1.0000	NA	
		meanlog	2.3773	1.3098	3.4449	0.5447	498.23	5.9827	1.3921	10.5734	2.3422	573.58
		sdlog	1.1679	0.8475	1.6095	0.1911		1.6395	0.9529	2.8210	0.4540	
	Non-mixture cure model (Gompertz)	theta	0.0916	0.0426	0.1863	NA		0.2228	0.1100	0.3992	NA	
		shape	0.2370	0.1506	0.3234	0.0441	507.75	0.1735	0.1067	0.2402	0.0341	576.35
		rate	0.0619	0.0395	0.0971	0.0142		0.0133	0.0073	0.0240	0.0040	
