# Supplementary material for: Cost-Effectiveness of Genomic Test-Directed Olaparib for Metastatic Castration-Resistant Prostate Cancer
Source: Front Pharmacol. 2021 Jan 26;11:610601. doi: 10.3389/fphar.2020.610601 (PMC7870786; doi:10.3389/fphar.2020.610601)
Supplement: Supplementary file 2 [file table2.pdf]

Appendix Table 2. Estimated parameters and AIC values from each survival model in MCRPC with at least 1 of the 15 prespecified gene alterations.

Strategies	Distributions	Parameters	PFS					OS				
			est	L95%	U95%	se	AIC	est	L95%	U95%	se	AIC
Standard care	Weibull	shape	1.1857	0.9051	1.5533	0.1634	173.67	1.4326	1.0546	1.9460	0.2239	228.18
		scale	5.7087	4.2412	7.6839	0.8655		17.4402	13.4734	22.5747	2.2962	
	Gamma	shape	1.5384	0.9919	2.3861	0.3445	171.71	1.7753	1.1212	2.8111	0.4163	227.44
		rate	0.2939	0.1622	0.5325	0.0891		0.1095	0.0591	0.2030	0.0345	
	Exp	rate	0.1722	0.1211	0.2449	0.0309	173.06	0.0527	0.0366	0.0758	0.0098	230.71
	Log-logistic	shape	1.8618	1.3965	2.4822	0.2732	164.48	1.8654	1.3745	2.5316	0.2906	226.43
		scale	3.5510	2.6666	4.7286	0.5189		12.7264	9.5861	16.8955	1.8399	
	Log-normal	meanlog	1.3264	1.0423	1.6105	0.1450	163.49	2.5523	2.2445	2.8602	0.1571	227.34
		sdlog	0.9053	0.6967	1.1763	0.1210		0.9588	0.7299	1.2594	0.1334	
	Gompertz	shape	-0.0515	-0.1828	0.0799	0.0670	174.42	0.0419	-0.0184	0.1023	0.0308	230.93
		rate	0.2002	0.1212	0.3307	0.0513		0.0372	0.0194	0.0716	0.0124	
	Royston/Parmar spline model (0 knot)	gamma0	-2.0655	-2.7130	-1.4181	0.3303	173.67	-4.0962	-5.3405	-2.8519	0.6349	228.18
		gamma1	1.1857	0.8655	1.5059	0.1634		1.4329	0.9940	1.8718	0.2239	
	Royston/Parmar spline model (1 knot)	gamma0	-3.3104	-4.4718	-2.1490	0.5926	157.82	-5.1911	-7.7041	-2.6781	1.2822	228.85
		gamma1	4.2553	2.3513	6.1592	0.9714		2.3420	0.5682	4.1159	0.9050	
		gamma2	1.0562	0.4672	1.6452	0.3005		0.1744	-0.1413	0.4901	0.1611	
	Royston/Parmar spline model (2 knot)	gamma0	-3.7016	-5.1713	-2.2320	0.7498	158.81	-4.6531	-7.0618	-2.2444	1.2290	229.85
		gamma1	6.1748	2.0978	10.2518	2.0801		1.6239	-0.3869	3.6348	1.0260	
		gamma2	3.0494	-0.6267	6.7256	1.8756		-0.7678	-2.4505	0.9149	0.8586	
		gamma3	-1.7568	-4.9670	1.4535	1.6379		1.2243	-1.0007	3.4492	1.1352	
	Mixture cure model (Weibull)	theta	0.2443	0.1307	0.4102	NA	161.48	0.2501	0.1046	0.4878	NA	228.15
		shape	1.9302	1.4545	2.5616	0.2787		1.7899	1.2511	2.5609	0.3271	
		scale	3.2917	2.6704	4.0575	0.3513		11.8378	8.5675	16.3565	1.9528	
	Mixture cure model (Gamma)	theta	0.2417	0.1275	0.4102	NA	159.25	0.2080	0.0563	0.5361	NA	228.22
		shape	3.3301	2.0109	5.5146	0.8570		2.2968	1.2619	4.1805	0.7018	
		rate	1.1395	0.6267	2.0717	0.3475		0.1981	0.0777	0.5048	0.0946	
	Mixture cure model (Exp)	theta	0.1441	0.0338	0.4472	NA	173.01	0.0004	0.0000	1.0000	NA	232.71
		rate	0.2452	0.1438	0.4180	0.0667		0.0527	0.0366	0.0760	0.0098	

	Mixture cure model (Log-logistic)	theta	0.2254	0.1085	0.4103	NA	158.60	0.1163	0.0046	0.7900	NA	228.13
		shape	2.8446	2.0195	4.0069	0.4972		2.0689	1.3122	3.2619	0.4806	
		scale	2.5176	1.9808	3.1998	0.3080		10.9610	6.5898	18.2319	2.8456	
	Mixture cure model (Log-normal)	theta	0.2276	0.1110	0.4104	NA	157.76	0.0011	0.0000	1.0000	NA	229.34
		meanlog	0.9403	0.7031	1.1774	0.1210		2.5508	2.2203	2.8813	0.1686	
		sdlog	0.5973	0.4427	0.8058	0.0913		0.9580	0.7244	1.2670	0.1366	
	Mixture cure model (Gompertz)	theta	0.2469	0.1338	0.4103	NA	166.67	0.2730	0.1412	0.4617	NA	230.17
		shape	0.4031	0.1717	0.6344	0.1180		0.1261	0.0434	0.2088	0.0422	
		rate	0.1334	0.0664	0.2680	0.0475		0.0359	0.0170	0.0757	0.0137	
	Non-mixture cure model (Weibull)	theta	0.2415	0.1290	0.4062	NA	160.32	0.2305	0.0708	0.5408	NA	228.06
		shape	2.1682	1.6374	2.8711	0.3106		1.9131	1.3027	2.8096	0.3751	
		scale	3.9936	3.0949	5.1532	0.5194		15.6790	8.4155	29.2118	4.9778	
	Non-mixture cure model (Gamma)	theta	0.2401	0.1258	0.4095	NA	158.46	0.1641	0.0206	0.6470	NA	228.21
		shape	3.5501	2.1606	5.8333	0.8995		2.2308	1.1950	4.1644	0.7105	
		rate	0.9883	0.4861	2.0094	0.3578		0.1227	0.0263	0.5711	0.0963	
	Non-mixture cure model (Exp)	theta	0.0207	0.0000	0.9925	NA	174.42	0.0000	0.0000	1.0000	NA	232.93
		rate	0.0516	0.0041	0.6531	0.0669		0.0026	0.0001	0.0792	0.0045	
	Non-mixture cure model (Log-logistic)	theta	0.2309	0.1139	0.4122	NA	158.90	0.1347	0.0143	0.6249	NA	228.09
		shape	2.7538	1.9356	3.9177	0.4953		2.0120	1.2614	3.2094	0.4793	
		scale	3.2448	2.3153	4.5474	0.5587		17.0511	6.5262	44.5497	8.3551	
	Non-mixture cure model (Log-normal)	theta	0.2230	0.1040	0.4150	NA	157.51	0.0018	0.0000	1.0000	NA	228.80
		meanlog	1.2059	0.8354	1.5765	0.1891		4.1980	-0.6029	8.9988	2.4495	
		sdlog	0.6326	0.4473	0.8945	0.1118		1.3413	0.5159	3.4872	0.6539	
	Non-mixture cure model (Gompertz)	theta	0.2458	0.1343	0.4065	NA	166.08	0.2702	0.1390	0.4591	NA	229.86
		shape	0.5182	0.2726	0.7637	0.1253		0.1561	0.0684	0.2438	0.0447	
		rate	0.0663	0.0306	0.1439	0.0262		0.0185	0.0080	0.0427	0.0079	
Olaparib	Weibull	shape	1.3371	1.1441	1.5626	0.1064	708.52	1.5160	1.2539	1.8328	0.1468	727.79
		scale	10.1910	8.8439	11.7432	0.7372		26.7885	23.0115	31.1855	2.0772	
	Gamma	shape	1.5404	1.2203	1.9444	0.1831	708.34	1.6329	1.2579	2.1198	0.2174	731.27
		rate	0.1610	0.1180	0.2197	0.0255		0.0620	0.0426	0.0902	0.0119	
	Exp	rate	0.0956	0.0791	0.1156	0.0092	718.31	0.0316	0.0255	0.0392	0.0035	741.45
	Log-logistic	shape	1.7102	1.4622	2.0001	0.1367	712.84	1.7709	1.4660	2.1391	0.1707	734.59
		scale	7.3260	6.1944	8.6643	0.6271		21.5753	18.2170	25.5528	1.8625	
	Log-normal	meanlog	1.9573	1.7797	2.1349	0.0906	714.91	3.1137	2.8877	3.3398	0.1153	753.16
		sdlog	1.0363	0.9030	1.1894	0.0728		1.1983	1.0208	1.4066	0.0980	
	Gompertz	shape	0.0681	0.0243	0.1118	0.0223	711.57	0.0680	0.0391	0.0969	0.0147	722.91
		rate	0.0649	0.0465	0.0907	0.0111		0.0142	0.0091	0.0222	0.0032	

	Royston/Parmar spline model (0 knot)	gamma0	-3.1031	-3.6174	-2.5887	0.2624	708.52	-4.9845	-5.8743	-4.0946	0.4540	727.79
		gamma1	1.3367	1.1283	1.5451	0.1063		1.5160	1.2283	1.8037	0.1468	
	Royston/Parmar spline model (1 knot)	gamma0	-3.1894	-3.8001	-2.5787	0.3116	710.21	-4.2170	-5.1065	-3.3276	0.4538	723.41
		gamma1	1.5007	0.8752	2.1262	0.3192		0.6787	0.1258	1.2316	0.2821	
		gamma2	0.0236	-0.0599	0.1070	0.0426		-0.1211	-0.2018	-0.0404	0.0412	
	Royston/Parmar spline model (2 knot)	gamma0	-3.1646	-3.7900	-2.5393	0.3191	710.17	-4.2791	-5.2142	-3.3441	0.4771	725.22
		gamma1	1.9582	1.0013	2.9151	0.4882		0.6402	0.0800	1.2004	0.2858	
		gamma2	0.3348	-0.1195	0.7891	0.2318		-0.2042	-0.9521	0.5437	0.3816	
		gamma3	-0.4262	-1.0435	0.1910	0.3149		0.1887	-1.1472	1.5246	0.6816	
	Mixture cure model (Weibull)	theta	0.0012	0.0000	1.0000	NA	710.53	0.0005	0.0000	1.0000	NA	729.79
		shape	1.3378	1.1429	1.5659	0.1075		1.5162	1.2540	1.8332	0.1469	
		scale	10.1719	8.7456	11.8309	0.7841		26.7758	22.9686	31.2140	2.0952	
	Mixture cure model (Gamma)	theta	0.0003	0.0000	1.0000	NA	710.35	0.0002	0.0000	1.0000	NA	733.27
		shape	1.5407	1.2203	1.9452	0.1833		1.6330	1.2579	2.1199	0.2174	
		rate	0.1611	0.1179	0.2201	0.0256		0.0620	0.0426	0.0903	0.0119	
	Mixture cure model (Exp)	theta	0.0001	0.0000	1.0000	NA	720.31	0.0001	0.0000	1.0000	NA	743.45
		rate	0.0956	0.0791	0.1156	0.0093		0.0316	0.0255	0.0392	0.0035	
	Mixture cure model (Log-logistic)	theta	0.0001	0.0000	1.0000	NA	714.85	0.0001	0.0000	1.0000	NA	736.59
		shape	1.7102	1.4623	2.0003	0.1367		1.7709	1.4660	2.1392	0.1707	
		scale	7.3248	6.1927	8.6637	0.6274		21.5747	18.2155	25.5532	1.8630	
	Mixture cure model (Log-normal)	theta	0.0002	0.0000	1.0000	NA	716.91	0.0004	0.0000	1.0000	NA	755.18
		meanlog	1.9572	1.7794	2.1349	0.0907		3.1133	2.8870	3.3397	0.1155	
		sdlog	1.0362	0.9028	1.1893	0.0729		1.1981	1.0206	1.4064	0.0980	
	Mixture cure model (Gompertz)	theta	0.0215	0.0000	0.9997	NA	713.55	0.2185	0.0695	0.5115	NA	723.81
		shape	0.0739	-0.0127	0.1604	0.0442		0.0933	0.0461	0.1404	0.0240	
		rate	0.0655	0.0463	0.0925	0.0115		0.0156	0.0096	0.0252	0.0038	
	Non-mixture cure model (Weibull)	theta	0.0000	0.0000	1.0000	NA	710.56	0.0000	0.0000	1.0000	NA	730.02
		shape	1.3589	1.1266	1.6392	0.1300		1.5320	1.2636	1.8574	0.1506	
		scale	88.0045	0.5775	13411.5315	225.6944		214.9319	35.1094	1315.7665	198.6899	
	Non-mixture cure model (Gamma)	theta	0.0000	0.0000	1.0000	NA	710.56	0.0000	0.0000	1.0000	NA	730.31
		shape	1.3952	1.1154	1.7452	0.1593		1.5467	1.2273	1.9491	0.1825	

		rate	0.0152	0.0004	0.5178	0.0274		0.0039	0.0000	653.1218	0.0242	
	Non-mixture cure model (Exp)	theta	0.0000	0.0000	1.0000	NA	721.04	0.0000	0.0000	1.0000	NA	744.07
		rate	0.0029	0.0004	0.0186	0.0027		0.0011	0.0000	0.0308	0.0018	
	Non-mixture cure model (Log-logistic)	theta	0.0000	0.0000	1.0000	NA	710.58	0.0000	0.0000	1.0000	NA	730.14
		shape	1.3685	1.1277	1.6608	0.1351		1.5358	1.2659	1.8633	0.1515	
		scale	108.1730	1.8945	6176.6400	223.2378		249.5811	36.1347	1723.8451	246.0875	
	Non-mixture cure model (Log-normal)	theta	0.0000	0.0000	1.0000	NA	710.80	0.0000	0.0000	1.0000	NA	736.97
		meanlog	6.8400	1.6517	12.0283	2.6472		8.7316	6.0572	11.4060	1.3645	
		sdlog	2.0915	1.3232	3.3059	0.4886		2.2126	1.7375	2.8176	0.2729	
	Non-mixture cure model (Gompertz)	theta	0.0167	0.0000	0.9994	NA	713.40	0.1998	0.0425	0.5841	NA	723.66
		shape	0.1022	-0.0242	0.2285	0.0645		0.1065	0.0516	0.1613	0.0280	
		rate	0.0151	0.0011	0.1990	0.0198		0.0072	0.0033	0.0155	0.0028	
