# Supplementary material for: Cost-Effectiveness of Genomic Test-Directed Olaparib for Metastatic Castration-Resistant Prostate Cancer
Source: Front Pharmacol. 2021 Jan 26;11:610601. doi: 10.3389/fphar.2020.610601 (PMC7870786; doi:10.3389/fphar.2020.610601)
Supplement: Supplementary file 3 [file table3.pdf]

Appendix Table 3. Other model inputs from PROfound trial.

| Parameter names | Standard care | Olaparib |
|---|---|---|
| Proportion of active regimens in the subsequent therapy | | |
| Docetaxel | 0.096 | 0.322 |
| Enzalutamide | 0.012 | 0.211 |
| Abiraterone | 0.024 | 0.178 |
| Cabazitaxel | 0.108 | 0.233 |
| Olaparib | 0.904 | 0.033 |
| Probability of adverse events (grade 1 and 2) | 0.500 | 0.440 |
| Probability of adverse events (grade⩾3) | 0.380 | 0.510 |
| Fatigue (grade⩾3) | 0.050 | 0.030 |
| Anemia (grade⩾3) | 0.050 | 0.210 |
| Vomiting (grade⩾3) | 0.008 | 0.023 |
| Backpain (grade⩾3) | 0.015 | 0.008 |
