# Supplementary material for: Cost-Effectiveness of Genomic Test-Directed Olaparib for Metastatic Castration-Resistant Prostate Cancer
Source: Front Pharmacol. 2021 Jan 26;11:610601. doi: 10.3389/fphar.2020.610601 (PMC7870786; doi:10.3389/fphar.2020.610601)
Supplement: Supplementary file 6 [file image2.pdf]

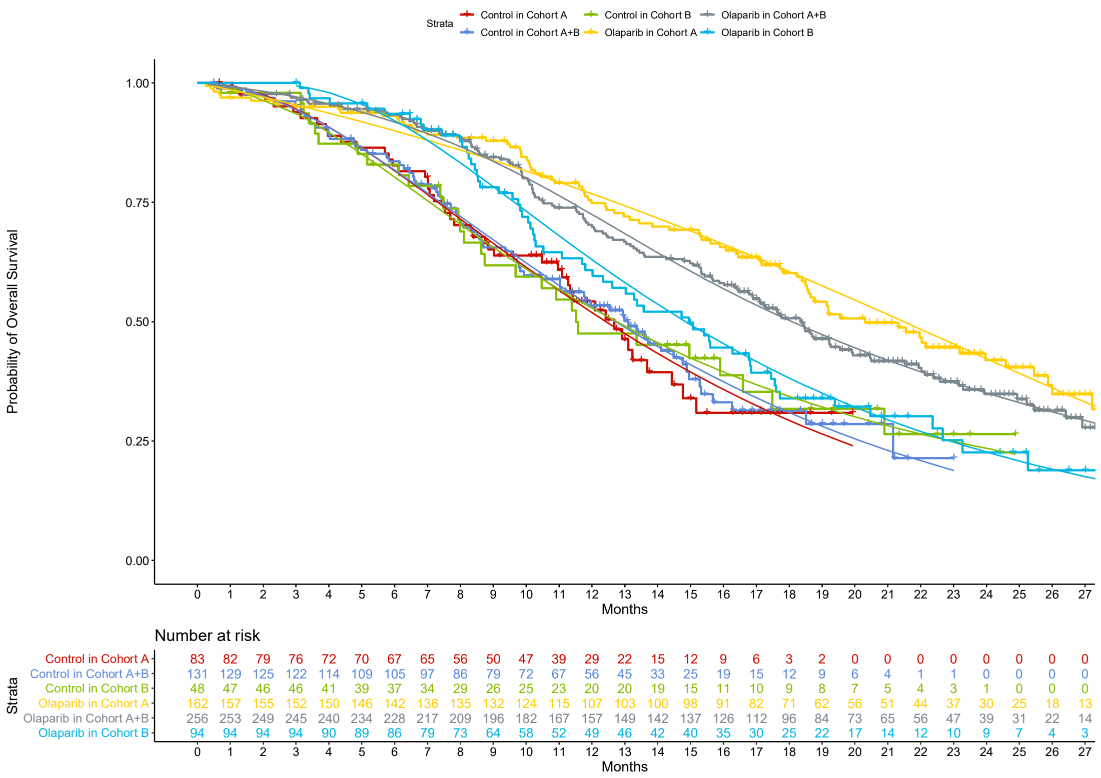

Appendix Figure 2: The replicated Kaplan–Meier OS curves of olaparib and standard care groups (control) in the cohort A, cohort B and cohort A+B of the PROfound 150 trial. The smooth lines indicated the survival curves predicting their corresponding best survival distributions. Patients with at least one alteration in BRCA1, BRCA2, or ATM were assigned to cohort A， regardless of co-occurring qualifying alterations in any of the other genes. Patients with alterations in any of the other 12 genes (BRIP1, BARD1, CDK12, CHEK1, CHEK2, FANCL, PALB2, PPP2R2A, RAD51B, RAD51C, RAD51D, and RAD54L) were assigned to cohort B. Cohort A+B included the men who had at least 1 of the BRCA1, BRCA2, ATM, BRIP1, BARD1, CDK12, CHEK1, CHEK2, FANCL, PALB2, PPP2R2A, RAD51B, RAD51C, RAD51D, and RAD54L gene alterations.
